# Magnetic susceptibility anisotropy of myocardium imaged by cardiovascular magnetic resonance reflects the anisotropy of myocardial filament α-helix polypeptide bonds

**DOI:** 10.1186/s12968-015-0159-4

**Published:** 2015-07-16

**Authors:** Russell Dibb, Yi Qi, Chunlei Liu

**Affiliations:** Center for In Vivo Microscopy, Duke University Medical Center, Box 3302, Durham, NC 27710 USA; Biomedical Engineering, Duke University Medical Center, Campus Box 90281, Durham, NC 27708 USA; Brain Imaging & Analysis Center, Duke University Medical Center, Box 3918, Durham, NC 27710 USA; Radiology, Duke University Medical Center, Box 3808, Durham, NC 27710 USA

**Keywords:** Anisotropic magnetic susceptibility, Myocardial fiber mapping, Multi-compartment relaxation, Resonance frequency shift

## Abstract

**Background:**

A key component of evaluating myocardial tissue function is the assessment of myofiber organization and structure. Studies suggest that striated muscle fibers are magnetically anisotropic, which, if measurable in the heart, may provide a tool to assess myocardial microstructure and function.

**Methods:**

To determine whether this weak anisotropy is observable and spatially quantifiable with cardiovascular magnetic resonance (CMR), both gradient-echo and diffusion-weighted data were collected from intact mouse heart specimens at 9.4 Tesla. Susceptibility anisotropy was experimentally calculated using a voxelwise analysis of myocardial tissue susceptibility as a function of myofiber angle. A myocardial tissue simulation was developed to evaluate the role of the known diamagnetic anisotropy of the peptide bond in the observed susceptibility contrast.

**Results:**

The CMR data revealed that myocardial tissue fibers that were parallel and perpendicular to the magnetic field direction appeared relatively paramagnetic and diamagnetic, respectively. A linear relationship was found between the magnetic susceptibility of the myocardial tissue and the squared sine of the myofiber angle with respect to the field direction. The multi-filament model simulation yielded susceptibility anisotropy values that reflected those found in the experimental data, and were consistent that this anisotropy decreased as the echo time increased.

**Conclusions:**

Though other sources of susceptibility anisotropy in myocardium may exist, the arrangement of peptide bonds in the myofilaments is a significant, and likely the most dominant source of susceptibility anisotropy. This anisotropy can be further exploited to probe the integrity and organization of myofibers in both healthy and diseased heart tissue.

## Background

Magnetic susceptibility describes the extent to which a substance becomes magnetized when placed in an external magnetic field. Many biological tissues exhibit either positive or negative susceptibility, and are termed paramagnetic or diamagnetic, respectively. Some of these tissues also have susceptibilities that are dependent on tissue orientation. In particular, striated muscle tissue is known to exhibit anisotropic magnetic susceptibility. Earlier studies have detected this susceptibility anisotropy in excised rabbit psoas muscle tissue suspended in a magnetic field [1] and in polymerized actin extracted from acetone powders of muscle tissue [2]. However, it is unknown if this anisotropy remains observable in bulk myocardium in an intact heart where the microstructures and molecular compositions are far more complex. Determining this anisotropy in an intact heart requires spatially localized susceptibility measurements, for which techniques relying on birefringence or superconducting quantum interference devices are limited.

Verifying the susceptibility anisotropy of myocardium and its underlying mechanisms using cardiovascular magnetic resonance (CMR) may lead to improved techniques for examining the microstructure of the heart. Myocardial fiber organization and structure are important determinants of myocardial stress and strain [3], are altered by cardiac hypertrophy [4] and infarction [5, 6], and are thought to play an important role in arrhythmogenesis [7]. Hence, mapping myofiber organization is important for assessing the functional properties of healthy and diseased hearts. Most commonly, ex vivo myocardial fiber mapping is carried out through diffusion tensor imaging (DTI) and histology [8–10]. Though a non-destructive imaging modality, cardiac DTI is challenged by spatial resolution limits and long scan times imposed by low signal-to-noise ratio (SNR), especially in the absence of contrast agent enhancement. On the other hand, histological techniques can yield whole-heart myofiber maps with very high spatial resolution, but are labor-intensive and require destruction of the organ. Alternatively, T_2_*-weighted gradient-recalled echo (GRE) CMR has shown potential as a method to visualize the microstructure of beating, isolated rat hearts [11]. In addition, GRE image phase is sensitive to changes in the magnetic field caused by magnetically susceptible components in tissues, such as deoxyhemoglobin, deoxymyoglobin, or calcification, and can be used for determining the susceptibility differences among tissues [12]. Considering the high-resolution capability of GRE phase and recent developments in susceptibility tensor imaging (STI) [13, 14], using CMR to image the susceptibility anisotropy of the heart may aid in assessing myocardial fiber integrity and alterations induced by cardiac diseases and disorders.

We present here a non-destructive method for imaging and quantifying the magnetic susceptibility anisotropy of whole myocardium *in situ* using CMR. The method, which details the relationship between apparent magnetic susceptibility and myofiber orientation, reveals that magnetic susceptibility anisotropy is extensive within the myocardium and not merely present in select tissue regions. Similar tools have recently been used to investigate anisotropic magnetic susceptibility in brain white matter [13, 15] and kidney tubules [16]. We hypothesize that the observed bulk magnetic susceptibility anisotropy of the heart originates from the α-helix polypeptides that are prevalent in myocardial filaments. We show by comparison of experimental and simulated results that the structurally organized [17] and diamagnetically anisotropic [18, 19] bonds forming these molecules are potentially the chief sources of the observed widespread anisotropy.

## Methods

### Animal model

All animal preparation protocols were approved by the Duke University Institutional Animal Care and Use Committee. Four adult, male C57BL/6 mice (Charles River Labs, Raleigh, NC) were anesthetized with pentobarbital (Nembutal, Lundbeck Inc., Deerfield, IL), and a catheter was inserted into the right jugular vein. Using a peristaltic pump, each animal was perfused first with 0.2 % heparin (1000 usp units/ml, Sagent Pharmaceuticals, Schaumburg, IL) in 0.9 % saline solution at a rate of 8 ml/min for 5 min. When perfusion began, the inferior vena cava and descending thoracic aorta were incised, allowing the blood to clear from the thorax, upper extremities and head. Next, the tissue was fixed using 150 ml of 10 % buffered formalin phosphate (SF 100–20, Fisher Scientific, Pittsburgh, PA) at a rate of 8 ml/min. Finally, to preserve the shape of the heart [20], the specimen was perfused with 1.3 % agarose gel (A9414-25G, Sigma-Aldrich, St. Louis, MO) at a rate of 8 ml/min for 2.5 min. The gel was allowed to solidify within the chambers of the heart for 25 min. The heart was then removed from the animal and stored for three days in 10 mM phosphate-buffered saline (pH 7.4, Sigma-Aldrich P-3813) prior to scanning.

### CMR microscopy

CMR experiments were performed using a 9.4 T (400 MHz) 8.9-cm vertical bore Oxford magnet controlled by an Agilent VnmrJ 4.0 console. Each myocardium specimen was firmly affixed in an 11-mm cylindrical polyethylene cartridge filled with Galden® (perfluoropolyether; Solvay Specialty Polymers) to provide a dark background in the images and mitigate tissue dehydration and susceptibility distortions at the specimen surface. In order to verify the presence of susceptibility anisotropy in localized myocardial regions, one heart specimen cartridge was placed inside a sphere, allowing for an arbitrary specimen orientation inside the coil. The coil apparatus [16] supported a solenoid radiofrequency resonator (21-mm diameter; 21-mm length). Magnitude and phase data were acquired using a GRE sequence with 16 echoes (TE_1_/ΔTE/TE_16_ = 2.2/4.2/65.2 ms, TR = 150 ms, α = 35°, array size = 400 × 300 × 300, isotropic voxel size = 45 μm, total scan time per orientation = 3.8 h). Prior to every image acquisition, the myocardium specimen was repositioned in a new orientation with respect to the magnetic field. Twelve image orientations were acquired for this particular specimen. To assess susceptibility anisotropy without the need to reorient the specimen, the other three heart specimens were scanned in a smaller, 12 mm × 25 mm (diameter × length) solenoid radiofrequency coil with the long axis of the myocardium fixed perpendicular to the main magnetic field direction. T_1_ recovery was measured in one of these specimens by acquiring image data from a central slice of the heart using a series of spin echo (SE) scans (TE = 10 ms; TR = 20, 40, 80, 160, 320, 640, 1280, and 2560 ms; array size = 128 × 128; resolution = 90 μm). MR magnitude and phase image data were acquired from each of the three specimens using a 3-D spoiled GRE sequence with 16 echoes (TE_1_/ΔTE/TE_16_ = 1.7/3.0/46.7 ms, TR = 200 ms, α = 35°, array size = 256 × 256× 256, isotropic voxel size = 45 μm, total scan time = 3.6 h). Diffusion tensor data were also acquired for all four individual specimens using one SE scan with b = 0 s/mm^2^ and 12 diffusion-encoded SE scans with diffusion time = 5.5 ms, pulse separation = 17.0 ms, and b = 1850 s/mm^2^ (TE = 23.6 ms, TR = 2000 ms, array size = 64 × 64 × 64, isotropic voxel size = 180 μm total scan time = 29.6 h). Due to the long T_1_ of tissue, the DTI protocol required the acquisition of a smaller array to achieve adequate signal-to-noise ratio and prevent overly long scan times.

### MR data reconstruction and processing

To extract the local susceptibility information from CMR images, the phase data must be appropriately processed to remove phase wraps as well as phase from sources outside the specimen. Thus, all of the multi-echo GRE image phase data were processed using an integrated Laplacian-based phase unwrapping and background phase removal algorithm, HARPERELLA [21]. With this processed phase information, the local susceptibility, **χ**, can then be approximated as a scalar-valued quantity by inverting the susceptibility-phase relationship in the laboratory frame of reference [13, 22],

1 $$ \upvarphi ={\mathrm{FT}}^{-1}\left\{\left(\frac{1}{3}-\frac{k_3^2}{{\mathbf{k}}^{\mathbf{T}}\mathbf{k}}\right)\mathrm{F}\mathrm{T}\left\{{\upchi}_{33}\right\}\right\}{\upgamma \upmu}_0HTE $$

where φ is the processed image phase, FT and FT^−1^ are the forward and inverse Fourier transforms, **k** is the spatial frequency vector, χ_33_ is the last term in the 3 × 3 magnetic susceptibility tensor, γ is the gyromagnetic ratio, μ_0_ is the vacuum permeability, *H* is the applied magnetic field strength, and *TE* is the echo time. Note that the χ_13_ and χ_23_ terms have been ignored in deriving Equation 1 assuming they are much smaller than χ_33_ [13]. This inversion problem is ill posed because the dipole kernel in Equation 1 is equal to zero when 3k_3_^2^ – **k**^**T**^**k** = 0. Several susceptibility mapping approaches have been formulated to overcome this challenge, including k-space thresholding [23], multiple-orientation sampling [24], nonlinear regularization [25], compressed-sensing estimation [26], and first-order derivative approximation [27]. In this study, the susceptibility maps were iteratively calculated using the LSQR method [27] because this algorithm computes susceptibility maps efficiently, requires only one sampling orientation, and offers a high quality solution. After the susceptibility maps were calculated for each individual echo, an average susceptibility map was calculated for each specimen from the multi-echo susceptibility data.

In order to match the GRE image data resolution of each specimen, the diffusion-weighted image data were either resampled to a 256^3^ array for the single-orientation data (*n* = 3 specimens), or a 400 × 300 × 300 array for the multi-orientation data (*n* = 1 specimen). A diffusion tensor map for each specimen was then estimated using Diffusion Toolkit [28]. A myofiber angle map was determined by calculating the angle between the major eigenvector of the diffusion tensor and the magnetic field direction vector. Lastly, tissue relaxation parameters were estimated for the single-orientation image data. A voxelwise calculation of the T_1_ relaxation time was made by fitting a longitudinal magnetization recovery curve to the signal from the 2D SE image data acquired using several TRs. The signal from each voxel in the multi-echo GRE magnitude image volumes was fit with a biexponential T_2_* decay function, S(t) = S_noise_ + S_0,GRE_[V_*i*_ × exp(−t / T_2i_*) + V_*e*_ × exp(−t / T_2e_*)], where S_noise_ is the noise floor of the magnitude signal and S_0,GRE_ is the signal at TE = 0 ms, assuming that the proton densities of the two signal pools are similar. This was done in order to acquire the intracellular and extracellular volume fraction (V_*i*_ and V_*e*_), as well as the intracellular and extracellular T_2_*. Using the mean (*n* = 3) volume fractions from the T_2_* decay fit, the signal data from the 2D image series of longitudinal magnetization recovery measurements were fit to the curve, S(t) = S_0,SE_[V_*i*_(1 – exp(−t / T_1i_*)) + V_*e*_(1 – exp(−t / T_1e_*))], subject to the constraint (V_*i*_ + V_*e*_) / T_1_ = V_*i*_ / T_1*i*_ + V_*e*_ / T_1*e*_. The peak values of the intra- and extracellular T_1_ distributions were used as rough estimates of the intra- and extracellular T_1_ relaxation times. Signal fitting was performed using the “fit” function in MATLAB R2014a (MathWorks, Natick, MA). All other computations were performed using MATLAB unless otherwise specified.

### Quantitative analysis of tissue susceptibility as a function of fiber angle

The multi-orientation GRE data were analyzed to determine the orientation dependence of magnetic susceptibility in myocardial tissue. First, all 12 GRE image volumes were registered to the corresponding diffusion tensor data. Three small (*n* = 10 pixels) regions of interest (ROIs) were manually selected from a 2-D image slice of the DTI data using the ITK-SNAP software [29]. Within each individual ROI were tissue voxels with similarly oriented myofibers, and the mean fiber direction of each ROI was approximately orthogonal to that of the two other ROIs. For each of the twelve registered GRE volume images, the mean apparent magnetic susceptibility of each ROI was related to the mean squared sine of the fiber angle with respect to the magnetic field. A linear regression model was then fit to the data in order to calculate the susceptibility anisotropy of myocardium.

Ideally, magnetic susceptibility anisotropy would be measured without the need to acquire data at multiple specimen orientations. For this reason, the correlation between susceptibility and myofiber orientation in the single-orientation image data was also studied. One large, 3D ROI was selected in each of the three remaining heart specimens. These ROIs included myocardial tissue but excluded the heart chambers as well as vessels that were large enough to segment. A voxelwise analysis then related the mean magnetic susceptibility of the multi-echo image data to the squared sine of the myocardial fiber angle in each specimen. Linear regression was again performed to measure the susceptibility anisotropy. Only voxels with an effective DTI fractional anisotropy above 0.3 and an intracellular voxel fraction, V_*i*_, within the middle 50 % of values were examined to ensure that the results included coherently oriented myofibers and excluded small vessels.

### A molecular and multi-filament model for susceptibility anisotropy

A myocardial tissue model was developed to evaluate the contribution of the diamagnetic anisotropy of the peptide bonds in myofilament proteins to the orientation-dependent magnetic susceptibility observed in the rodent heart. Within a sarcomere is an organized lattice of thick and thin myofilaments. These filaments are primarily constructed out of the proteins myosin, tropomyosin, and actin. Both myosin and tropomyosin predominantly contain polypeptide chains in the α-helix form, which is known to be diamagnetically anisotropic due to the structure of its peptide bonds [18]. In myosin, two α-helices wind together, forming a coiled-coil structure. This myosin “tail” then twists together with several other myosin tails to form the thick filament. Like the myosin tail, tropomyosin is a coiled-coil protein, with two tropomyosin strands running along the thin filament. The other major component of the thin filament is actin, which contains about 40 % α-helix [30]. Within the A-band of the sarcomere, overlapping myosin, tropomyosin, and actin filaments are oriented parallel to each other and to the long axis of the myofiber.

**Fig. 1:**
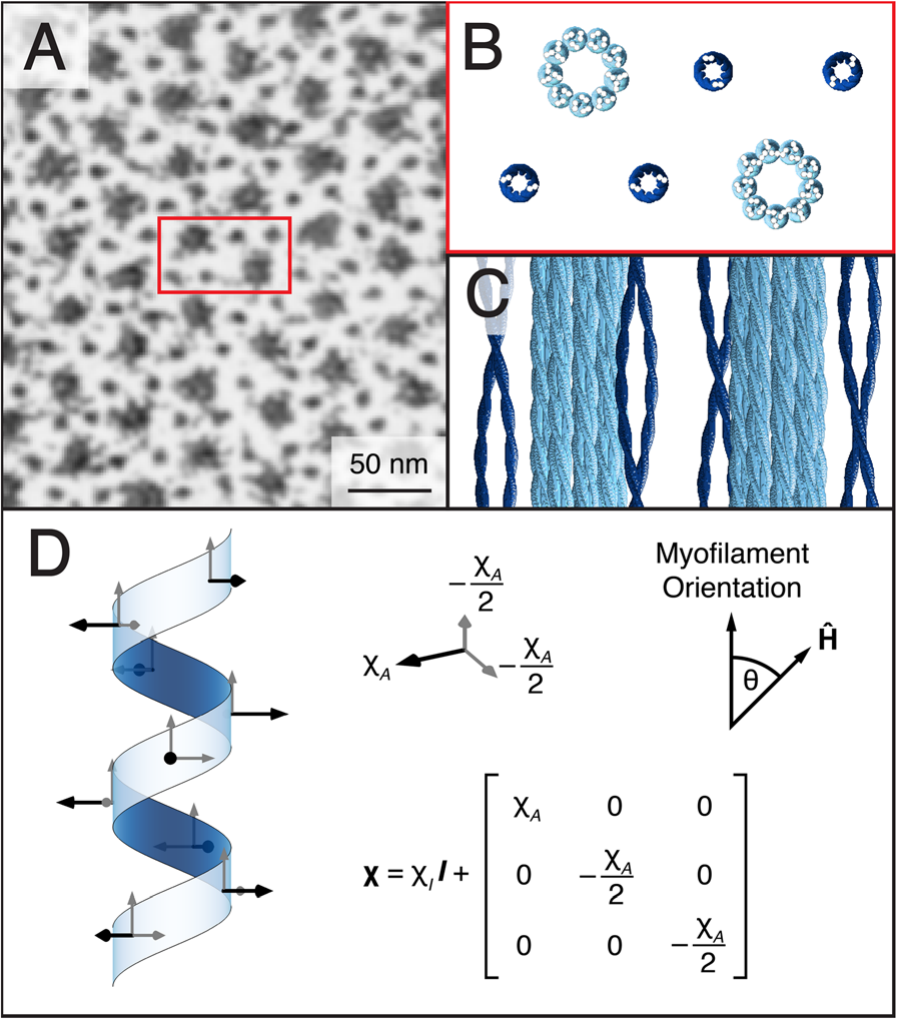
Microstructural and mathematical basis of the myofilament model. **a** An electron microscope image of a cross-section of the sarcomere highlights the myofilament lattice in myocardial tissue (image courtesy of Margaret Goldstein, PhD, Baylor College of Medicine and Robert Perz-Edwards, PhD, Duke University). **b** A cross-sectional rendering of the myofilament model volume represents a unit that repeats throughout the sarcomere. **c** Thick (*light blue*) and thin (*dark blue*) filaments are represented in the model volume by secondary and tertiary α-helical structures. The F-actin is not represented in the model volume due to the less coherent organization of α-helices within actin subunits. **d** Bond locations and susceptibility tensors are defined according to the molecular structure of the α-helix. There is an average of 3.6 peptide bonds per helix repeat

A section of sarcomere A-band was simulated inside a 69.3 × 40.0 × 42.9 nm volume and was divided into a 462 × 266 × 286 voxel array with 0.15 nm isotropic resolution. This resolution was selected because it is the axial spacing between peptides in the α-helix and is similar to the length of the diamagnetically anisotropic peptide bond (0.132 nm) [17]. Two thick and four thin filaments were simulated in the volume according to their α-helical, secondary, and tertiary structures [31, 32], and then arranged to mimic the lattice structure of the smallest repeatable unit of the sarcomere (Fig. 1a-c). The simulated volume maintains the 2:1 thin-to-thick filament ratio observed in vertebrate myocardium with thick and thin filaments coaxially spaced at 40.0 and 23.1 nm, respectively [33]. Due to the less coherent organization of the α-helices in actin subunits [34], only myosin and tropomyosin α-helices are represented in the volume (Fig. 1b-c). Finally, the peptide bond locations were simulated in the volume according to the pitch and spacing of the α-helix polypeptide backbone. Table 1 details the literature-derived myofilament microstructural parameters used in the simulation.

**Table 1:** Microstructural parameters defining the myofilament lattice in the model volume

Microstructural Feature	Diameter, nm	Pitch, nm
Thick filament	16.0 [32]	-
Thick subfilament	4.0 [32]	42.9 [61]
Myosin tail	2.0 [62]	4.3 [63]
Thin filament	7.0 [64]	77.0 [65]
Tropomyosin tail	2.0 [66]	3.7 [65]
α-helix	1.0 [67]	0.54 [67]

When amino acids are arranged in an α-helix, the peptide bonds all lie in a plane parallel to the helix axis (Fig. 1d). The magnetic susceptibility normal to this plane is defined as the anisotropic susceptibility component, χ_*A*_. For the purposes of this model, the in-plane susceptibilities are approximately equal, i.e., the susceptibility tensor is axially symmetric about the out-of-plane axis of the bond. This assumption is made because formamide, a simple prototype of a peptide linkage [35], is almost axially symmetric with respect to its magnetic susceptibility components about the out-of-plane (and most diamagnetic) axis [36]. Each voxel within the volume that contains a peptide bond was assigned the same *relative* susceptibility tensor, **χ**, reflecting the axial magnetic susceptibility symmetry of the peptide group (Fig. 1d). Each of these tensors was then rotated according to its position in the α-helix. Using the theoretical molar susceptibility anisotropy value calculated by Pauling, −5.36 × 10^−6^ cm^3^/mol (CGS units) [19], and the volume of a simulated voxel, the model-specific volume susceptibility anisotropy of each voxel containing a peptide group was calculated as Δχ = χ_11_ – (χ_22_ + χ_33_) / 2 = −33.14 ppm (SI units). From this value, the anisotropic susceptibility component is then χ_*A*_ = 2Δχ / 3 = −22.09 ppm. The isotropic susceptibility value of the peptide group, χ_*I*_, is not fixed as it is evaluated relative to the reference susceptibility. All other voxels in the simulated volume were assigned a relative susceptibility tensor with value **0**.

With the axes of the simulated fibers oriented at an angle (θ) to the direction of the static field (**Ĥ**), three-dimensional frequency maps for the model array (*f*_array_) were simulated using the tensor formulation of the susceptibility-frequency equation in the subject frame of reference [13],

2 $$ {f}_{\mathrm{array}}={\mathrm{FT}}^{-1}\left\{\left(\frac{1}{3}{\widehat{\mathbf{H}}}^{\mathbf{T}}\mathrm{F}\mathrm{T}\left\{\boldsymbol{\upchi} \right\}\widehat{\mathbf{H}}-{\widehat{\mathbf{H}}}^{\mathbf{T}}\mathbf{k}\frac{{\mathbf{k}}^{\mathbf{T}}\mathrm{F}\mathrm{T}\left\{\boldsymbol{\upchi} \right\}\widehat{\mathbf{H}}}{k^2}\right)\mathrm{F}\mathrm{T}\left\{\boldsymbol{\upchi} \right\}\right\}{\upgamma \upmu}_0H $$

Finally, a complex average of uniformly distributed spins across the entire 3D frequency map yielded a single, complex-valued signal to represent the intracellular volume. In contrast, the extracellular volume was assigned a relative susceptibility tensor value of **0**, which is the reference susceptibility. As a result, the net signal of the extracellular space has zero phase.

Within the myocardium are two anatomical compartments with distinct signal contributions: intracellular and extracellular [37]. To simulate a model susceptibility value for a volume the size of a voxel in the experimental data, the complex-valued signal from the myofilament lattice in the intracellular compartment must be appropriately combined with the signal from the extracellular compartment. In this model, the magnetic resonance signal frequency shift, Δ*f*, generated by a myocardial tissue voxel is the result of a weighted average:

3 $$ \varDelta f\kern0.5em =\angle \left\{{\sum}_{j=i,e}{S}_j{\uprho}_j{V}_j{e}^{-TE/{T}_{2_j}^{\ast }}\frac{\left(1-{e}^{-TR/{T}_{1_j}}\right) \sin \kern0ex \upalpha}{1-{e}^{-TR/{T}_{1_j}} \cos \kern0ex \upalpha}\right\}/-2\uppi TE $$

Here, the subscripts *i* and *e* represent the intra- and extracellular compartments, respectively; *S* is the complex-valued signal; ρ is the relative spin density, which the model assumes is the same for each compartment for simplicity since the literature values for the water content of perfused rodent hearts varies by study [38]; and α is the flip angle. According to Equation 3, the model applies T_1_ and T_2_* weighting to the intra- and extracellular compartments, but also takes into account the intracellular T_2_’ decay that is inherently simulated by the model due to the inhomogeneous magnetic susceptibility distribution of the intracellular volume.

An estimate of the bulk susceptibility of a voxel containing myocardial tissue, χ, was then calculated using the theoretical relationship Δ*f* / *f*_0_ = χ / 3 with the correction of spherical inclusion [39], where *f*_0_ is the resonance frequency. The simulation was then repeated for a range of myocardial fiber orientations (0–90°) with respect to **Ĥ** to model susceptibility anisotropy as a function of TE. The isotropic susceptibility value of the peptide bond tensor, χ_*I*_, was calculated by the model to fit the specimen susceptibility data (which is a relative measure and not absolute) using a mean squared error minimizing algorithm that was weighted by the inverse of the standard deviation of the experimental data (step size = 0.01 ppm). With χ_*I*_ calculated, the simulation was then repeated while excluding the extracellular space from the model in order to estimate the volume susceptibility anisotropy of the myofilament lattice. The thick and thin filaments were then simulated separately to ascertain the relative contributions of each structure to the tissue susceptibility anisotropy. Variations of this model were simulated to analyze the impact of implementing multi-compartment signal relaxation on the model volume’s bulk susceptibility anisotropy. The first scenario involved omitting T_1_, and then both T_1_ and T_2_* relaxation from the model. In the second scenario, the T_2_* weighting was adjusted by superposing T_2_, T_2_* and no additional weighting on top of the inherent intracellular T_2_’ that exists due to the field inhomogeneity produced by the simulated peptide bonds.

## Results

### Orientation-dependent susceptibility contrast

**Fig. 2:**
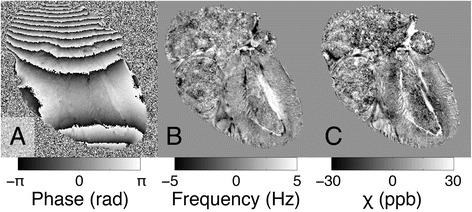
Reconstruction pipeline for processing the magnetic susceptibility of the mouse heart specimens. Raw phase (**a**), frequency (**b**), and magnetic susceptibility (**c**) maps were generated for each echo from multi-echo gradient-recalled echo image data. Shown are examples of results at TE = 22.7 ms

**Fig. 3:**
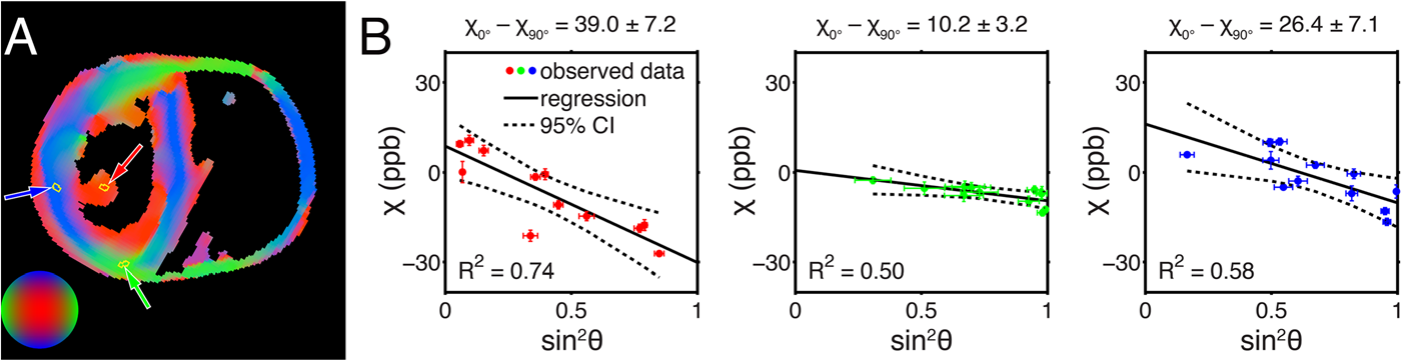
Mean apparent magnetic susceptibility as a function of mean myofiber orientation in localized tissue regions. Myofiber orientation was calculated from the principal eigenvector of the DTI data (**a**). Each small ROI (*n* = 10 voxels) is outlined in yellow and represents myocardial fibers from one of three approximately orthogonal directions (represented by green, red, and blue). Magnetic susceptibility data were calculated from multi-orientation GRE image data and correlated with the fiber orientation. Each data point represents a measurement acquired from an individual specimen orientation. The results of the linear regression, as well as a 95 % confidence interval, are given for each region (**b**). The susceptibility anisotropy estimates (mean ± standard error) for each linear fit are shown. Error bars represent the standard deviations of each parameter within an individual ROI

**Fig. 4:**
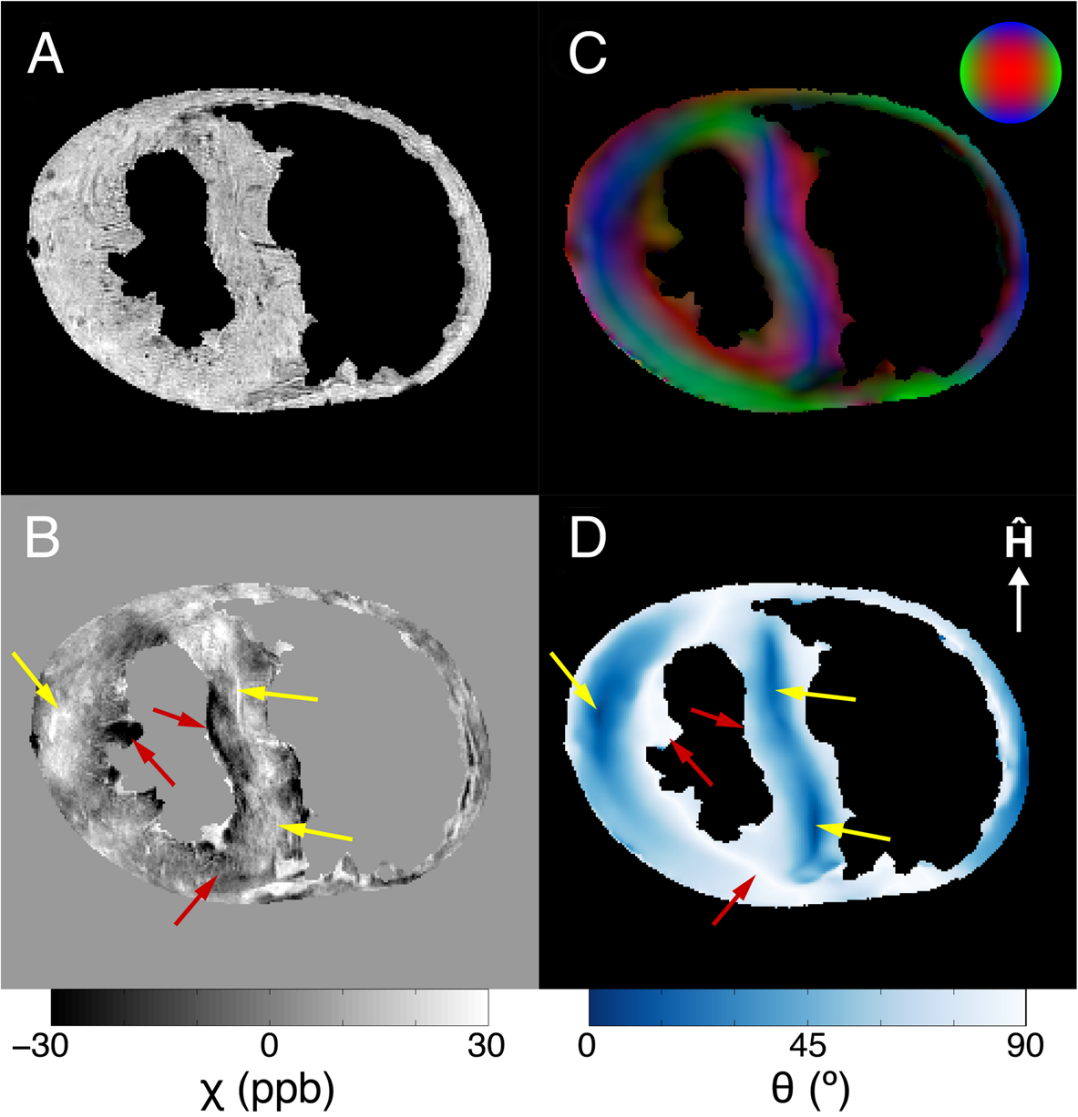
Correlation between apparent magnetic susceptibility and myofiber orientation in a typical specimen. Anatomical reference image created by averaging GRE magnitude data from echoes 1–4 (**a**). Mean magnetic susceptibility map calculated from multi-echo GRE image phase data (**b**). DTI myofiber orientation map that has been weighted by the fractional anisotropy and converted into red/green/blue values, where blue indicates the magnetic field direction (**c**). The major eigenvector of the DTI data was used to calculate the myofiber angle relative to the magnetic field direction (**d**). Yellow arrows indicate myofibers that are nearly parallel to B_0_ and more paramagnetic. Red arrows indicate myofibers that are nearly perpendicular to B_0_ and more diamagnetic

The results of the phase processing pipeline for a single echo image from a typical myocardium specimen are shown in Fig. 2. Since the gel-filled regions of the heart have a tendency to obfuscate image properties exhibited by myocardial tissue (Fig. 2b-c), the chambers of the heart have been masked out for display in the figures that follow. The relationship between the mean magnetic susceptibility contrast and the mean myofiber orientation for the multi-orientation GRE data is shown in Fig. 3. Linear regression reveals a negative trend in each of the three small ROIs, though the degree of the susceptibility anisotropy and the coefficient of determination vary. The correlation is strongest in the papillary muscles (red), which, not surprisingly, also have very strong DTI fractional anisotropy. The relationship between the mean magnetic susceptibility contrast of the multi-echo GRE image data acquired with a single-orientation and the DTI-based myofiber orientation of the tissue is illustrated for a typical specimen in Fig. 4. As seen in the mean susceptibility (Fig. 4b) and myofiber angle (Fig. 4d) maps, myofibers perpendicular to the field appear diamagnetic, whereas those parallel to the field appear paramagnetic relative to the reference susceptibility.

**Fig. 5:**
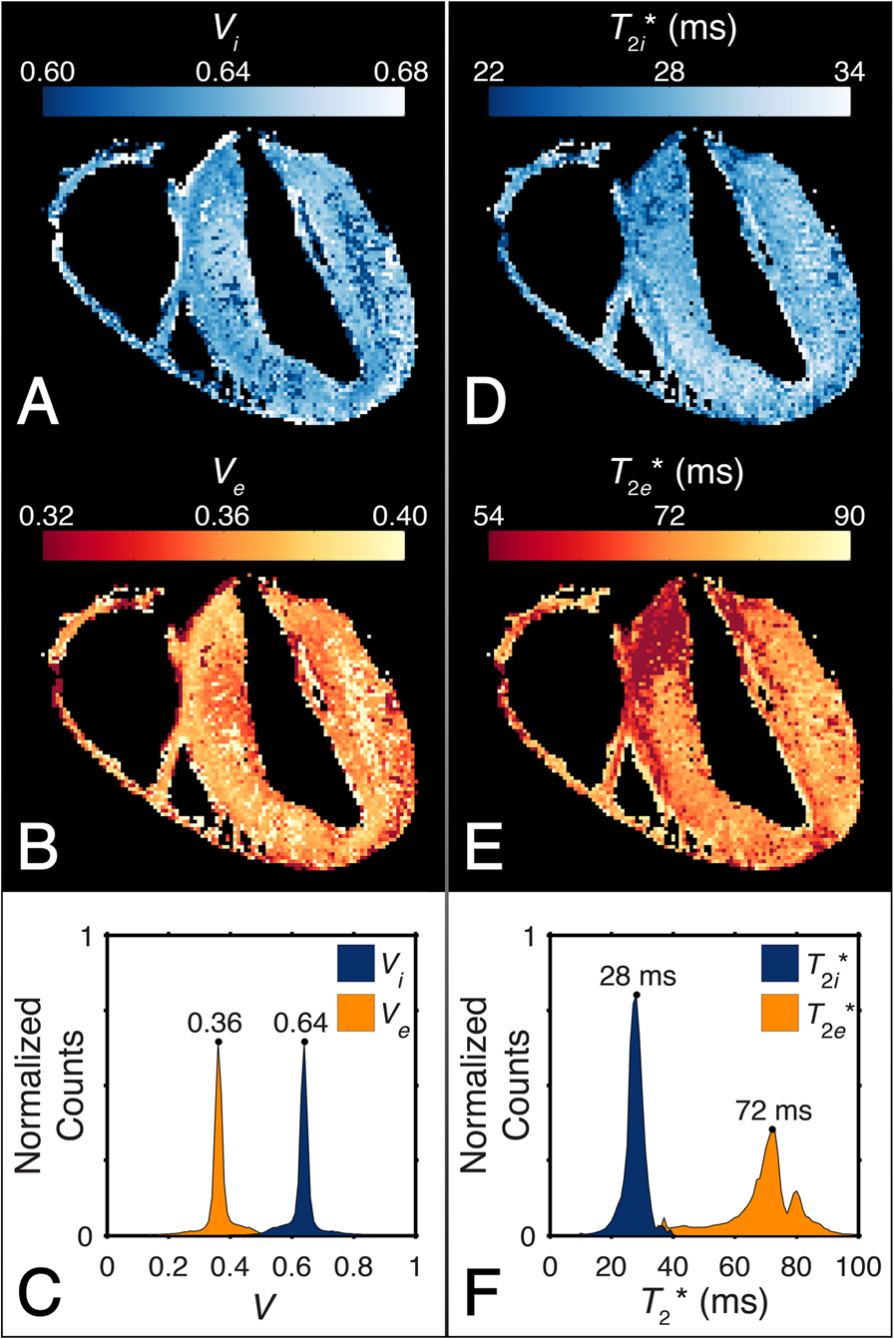
Myocardium compartmentalization. The results of the voxelwise (*n* = 1,515,232), SNR-weighted, biexponential signal-fitting algorithm used to probe compartment volume fractions and T_2_* values in a typical specimen are shown in the intracellular (**a**) and extracellular (**b**) volume fraction maps and their respective distributions (**c**). Intracellular (**d**) and extracellular (**e**) T_2_* calculations yield T_2_* distributions (**f**) with well-defined peaks. The modal volume fraction and T_2_* values for the two compartments are annotated within the figure with the subscripts *i* and *e* indicating intra- and extracellular compartments, respectively

**Fig. 6:**
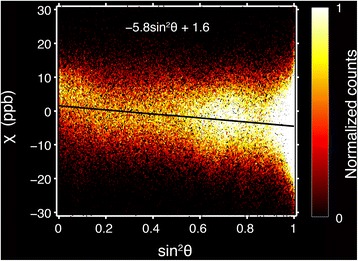
Magnetic susceptibility anisotropy of myocardium. Two-dimensional histogram of the voxelwise (*n* = 532,754) mean magnetic susceptibility as a function of the squared sine of the myofiber angle for a typical specimen. The average apparent magnetic susceptibility was calculated from the multi-echo GRE data acquired in a single specimen orientation. Myofiber orientation was calculated from the principal eigenvector of the DTI data

The results of the biexponential signal-fitting algorithm from a typical heart specimen are shown in Fig. 5. Histograms detailing the distribution of volume fractions and T_2_* relaxation times in the myocardium were also generated (Fig. 5c,f). Based on these histograms, the peak intra- and extracellular compartment volume fractions (V_*i*_ and V_*e*_) and T_2_* values were calculated for each specimen. The peak T_1_ value of each compartment’s distribution was then calculated. These volume fractions and relaxation properties are listed in Table 2. Using the volume fraction maps to segment out tissue voxels that were likely to contain a large proportion of small vessels, a qualitative analysis then related the magnetic susceptibility to the squared sine of the myocardial fiber angle in the resulting tissue ROI. Similar to the qualitative results in Fig. 4, the voxelwise analysis reported that the magnetic susceptibility of muscle tissue appears more diamagnetic as the myocardial fiber angle increases. For a typical specimen, this is emphasized by the linear regression fit of the mean magnetic susceptibility of the multi-echo GRE data as a function of sin^2^θ in Fig. 6. From the three regression fits (mean R^2^ = 0.035), the estimated susceptibility anisotropy (χ_0°_ – χ_90°_) was 5.94 ± 0.47 ppb, and the estimated susceptibility of field-parallel fibers (χ_0°_) was 0.28 ± 1.95 ppb. The weak correlation signifies that susceptibility anisotropy varies throughout the tissue, but is still measurably present in the heart as a whole.

**Table 2:** Intra- and extracellular relaxation properties of *ex vivo* myocardium (mean ± standard deviation)

Parameter	Intracellular	Extracellular
Volume fraction, *n* = 3	0.65 ± 0.01	0.35 ± 0.01
T_2_* (ms), *n* = 3	29 ± 1	76 ± 4
T_1_ (ms), *n* = 1	1050	1750

### Model prediction of susceptibility anisotropy

**Fig. 7:**
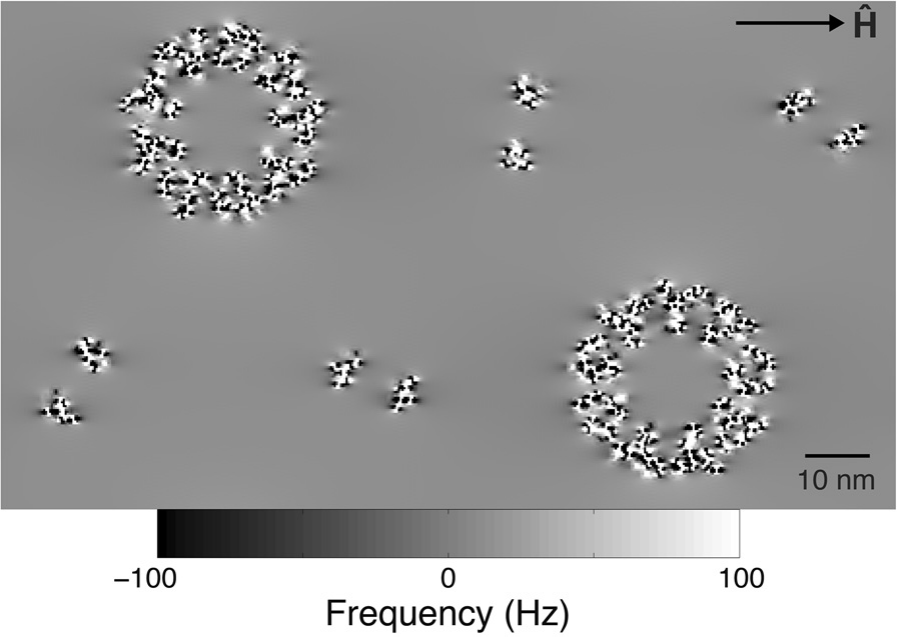
A simulated frequency map at 9.4 T calculated with the myofilament orientation perpendicular to Ĥ. For this and all myofilament orientations, the spacing between myofilaments was sufficient that the effects of individual myofilaments on the frequency offset were almost completely isolated

A sample image of the forward calculation of the frequency map from the susceptibility tensor-valued myocardial tissue volume array is shown in Fig. 7. Individual myofilaments within the simulated volume yield frequency contributions that are predominantly isolated from one another. Thus, if the size of the model volume were increased to include multiple simulated volume units, measurements of susceptibility anisotropy would be consistent with a single, simulated volume unit.

**Fig. 8:**
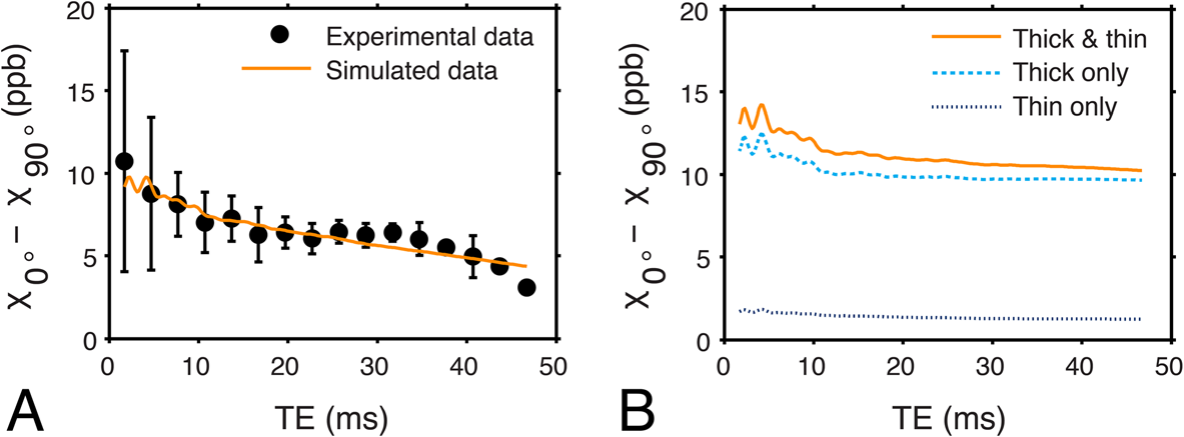
Comparison between experimental and simulated susceptibility anisotropy data. The experimental data were acquired from whole mouse myocardium specimens (*n* = 3) at 9.4 T, whereas the simulated data were calculated using the multi-filament model with two signal compartments (**a**). The simulated anisotropy of the intracellular compartment (**b**) is calculated by ignoring signal contributions from the extracellular compartment and using the fitted isotropic component (χ_*I*_) and fixed anisotropic (χ_*A*_) component values of the peptide group susceptibility tensor. The simulated anisotropy data are shown for when the model’s intracellular compartment contains both thick and thin filaments, thick filaments only, and thin filaments only. Error bars indicate the standard deviation of the experimental data

Using the bulk susceptibility anisotropy data calculated from individual GRE echo images, the model computed a best-fit relative isotropic susceptibility value for the peptide group tensor (χ_*I*_ = 0.04 ± 0.01 ppm). Fig. 8a compares the simulated anisotropy data to the data acquired from the mouse heart specimens using CMR. As TE increases, the susceptibility anisotropy decreases in both the simulated and acquired data. Figure 8b shows that the simulated susceptibility anisotropy of the intracellular compartment decreases as TE increases even though the extracellular compartment is being ignored. This is likely due to the dephasing of spins within the volume, which intensifies as TE increases.

**Fig. 9:**
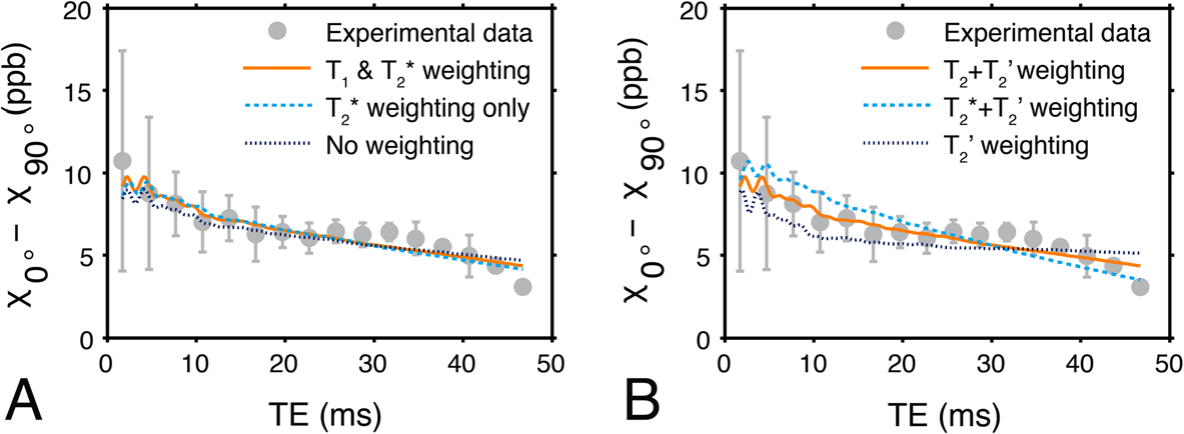
Comparison between susceptibility anisotropy data simulated under different signal models. **a** Experimental susceptibility anisotropy and simulated susceptibility anisotropy data when the model includes T_1_ and T_2_*-weighting, T_2_*-weighting only, and no weighting in both the intra- and extracellular signal compartments. The simulated anisotropy is minimally affected by excluding multi-compartment relaxation effects from the model. **b** Experimental data from the specimen and simulated data from a model demonstrating the effect of superposing T_2_, T_2_*, and no additional weighting on top of the inherent intracellular T_2_’ that exists due to the field inhomogeneity produced by the simulated peptide bonds. The additional intracellular spin-spin relaxation weighting significantly impacts susceptibility anisotropy as a function of TE, and is most correct in the model simulating T_2_’ superposed with T_2_. Error bars indicate the standard deviation of the experimental data

The results of the simulation for the different models of multi-compartment relaxation are shown in Fig. 9. Both multi-compartment T_1_ and T_2_* relaxation have a relatively small effect on susceptibility *anisotropy* as a function of TE, though the fitted *isotropic* susceptibility value of the peptide group differs for the data that was only T_2_* weighted (χ_*I*_ = −0.8 ± 0.1 ppm) and not weighted (χ_*I*_ = 0 ± 0.1 ppm). This demonstrates that, even if relaxation time estimates are only approximate, the model still reasonably simulates the experimental susceptibility anisotropy data.

To emulate the experimental data acquired from the specimen, the model applied T_2_*-weighting to the simulated extracellular compartment signal magnitude to represent the effect of spin dephasing. In the intracellular compartment, however, T_2_’-weighting is inherently modeled due to the inhomogeneous magnetic susceptibility distribution of the simulated intracellular volume. The effective simulated intracellular T_2_’ value associated with the mean myocardial fiber angle of the three specimens (~50°) was T_2*i*_’ = 40 ms. Thus, it was necessary to superpose T_2_-weighting on the intracellular volume signal to effectively produce T_2_* signal weighting. In the complete model, T_2_-weighting was applied to the intracellular compartment with T_2*i*_ = 109 ms in order to enforce the condition that 1/T_2i_* = 1/T_2*i*_ + 1/T_2*i*_’. Figure 9b emphasizes the effect of erroneously replacing T_2_-weighting with either T_2_*-weighting, or no additional weighting on top of the inherent T_2_’ signal weighting in the intracellular compartment. The fitted isotropic susceptibility for these model variations were χ_*I*_ = −1.2 ± 0.1 (additional T_2_*-weighting) and χ_*I*_ = 1.5 ± 0.1 ppm (no additional weighting).

## Discussion

### Multi-filament model predicts susceptibility anisotropy

Myocardial fiber orientation plays an essential role in heart structure and function, and is frequently assessed in studies of healthy and diseased myocardium. Susceptibility imaging may potentially be used to analyze the organization and integrity of myocardial fibers by exploiting the anisotropic magnetic susceptibility of muscle tissue. The results produced by this microstructural model show that the diamagnetic anisotropy of the polypeptide bonds in muscle filaments is potentially a major contributor to the fiber-orientation-dependent susceptibility contrast visualized using CMR. This is because the amino acids in the α-helix are arranged with the peptide groups lying in a plane parallel to the helix axis. The summation of the individual peptide bond susceptibilities, which each have their most diamagnetic component perpendicular to the myofiber axis, produces bulk magnetic anisotropy on a scale measurable by susceptibility CMR. To simulate CMR data that exhibited susceptibility anisotropy using a model of the magnetically anisotropic α-helix, it was necessary to calculate a fitted isotropic susceptibility value for the peptide group tensor. Magnetic susceptibility values are calculated from image frequency data that are referenced to the carrier frequency of excitation radiofrequency pulses, hence, the isotropic susceptibility component of the peptide group tensor is relative and may vary from scan to scan.

### Multi-compartment effects and applications

The intra- and extracellular population fractions calculated for the short-T_2_* and long-T_2_* compartments (Table 2) are quite similar to the values found in other studies of excised rodent myocardium [37]. We acknowledge that not all of intracellular space is composed of the myofilament lattice. The myofibril volume fraction has been measured at 0.52 ± 0.04 of total perfused mouse myocardium cell volume [40]. For the purposes of maintaining a two-pool model, however, the simplifying assumption was made that the myofibril volume fraction was equal to the volume fraction of the short-T_2_* compartment. Based on our observations from the model volume simulation, multi-pool relaxation strongly affects susceptibility contrast and anisotropy in muscle tissue, as has been suggested by similar models for central nervous system white matter [41, 42]. This relaxation is intensified by contrast agents, which also augment susceptibility anisotropy in brain white matter [42], kidney tubules [16], and potentially the myocardium, though the underlying mechanism is unclear. Earlier studies have shown that compartmentalization of Gd-DTPA in the extracellular space of muscle tissue yields a significant T_2_ reduction in extracellular water, but not intracellular water [43]. In this case, Gd may cause significant spin dephasing and signal attenuation in the extracellular compartment, leaving the anisotropic intracellular compartment to dominate the measured tissue susceptibility. Alternatively, the increased anisotropy may be induced by a local magnetic field generated by the contrast agent in the organized tissue matrix. Though the mechanisms are not yet understood, using contrast agents to enhance susceptibility anisotropy is likely to improve the feasibility of STI of the myocardium. Applying STI to the study of animal models of myocardial disease will help verify the source(s) of myocardial susceptibility anisotropy.

In addition to contrast agents, tissue pH and the use of fixatives during specimen preparation may complicate analyses of multi-compartment signal relaxation. It has been shown in skeletal muscle that decreases in intracellular pH due to the accumulation of the end-products of anaerobic metabolism during exercise correlate with an increased intracellular T_2_ relaxation time [44]. Changes to intra- or extracellular signal T_2_ relaxation times associated with pH would affect the weighting of the signal phase as described by Equation 3. The impact of this weighting would be very small, but it may explain a fraction of the decrease in the measured anisotropy at extremely long TEs. However, substantial and spatially coordinated pH differences are not expected in fixed tissue specimens that have soaked for days in phosphate-buffered saline solution.

Following the extraction of the heart specimen from the mouse, the tissue begins to degrade very rapidly. Due to the technical challenges of scanning fresh tissue for extended periods of time, particularly when acquiring data in multiple specimen orientations, it was determined that *ex vivo* data acquisition would require tissue fixation. Fixatives are known to shorten T_1_ and T_2_ in both nervous and cardiac tissues [45, 46], though the effects on T_2_ are reversible by soaking the specimen in phosphate-buffered saline [46]. The simulated data suggest that changes to the T_1_ weighting would affect the isotropic susceptibility but have minimal impact on the susceptibility anisotropy measurements (Fig. 9a). This is especially true if the applied fixative shortens the T_1_ of the intra- and extracellular signal compartments in a relatively proportional manner. Still, this is a limitation to the current study, particularly since fixative use is reserved for the preclinical domain. An alternative specimen preparation might exclude fixatives but require the inclusion of contrast agent in order to shorten the scan time and lessen the impact of tissue degradation. However, the presence of paramagnetic contrast agent in the specimen would complicate measurements of myocardial susceptibility anisotropy. This is because contrast agents like Gd-DTPA are more highly concentrated in the extracellular volume of muscle tissue [43], thereby disproportionally shortening both the longitudinal and transverse relaxation times and artificially inflating the susceptibility of the extracellular volume.

### Data acquisition and analysis considerations

One goal of this study was to measure the inherent susceptibility anisotropy of myocardium, i.e., without the use of contrast agents. As a result, SNR limitations were a factor in both the DTI and GRE data acquisition and analysis. To achieve reasonable scan times, the DTI data were acquired at a lower resolution, 180 μm, relative to the GRE data resolution, 45 μm (see *Methods*). Myocardial tissue is laminar, and has a typical tissue layer width of 100 μm. As a result, some partial voluming of the diffusion signal is expected. However, because the orientation of myocardial tissue fibers varies smoothly from layer to layer, the interpolated DTI data reasonably describe myofiber orientations in the heart at the higher resolution (Fig. 4). As for the GRE data, the first two echo images from each specimen have very low phase SNR, which is expected when TE < < T_2_* [47]. Measurements of myocardial susceptibility anisotropy at short TEs vary greatly, which explains the data acquired at TE < 5 ms (Fig. 8).

Another goal of this study was to measure the average magnetic susceptibility anisotropy using data from a single specimen orientation. This estimation, however, did not quantify the tensor-valued susceptibility on a voxel basis and might be confounded by the spatial heterogeneity of myofibers. Unlike the multi-orientation data acquisition, which allowed for the analysis of the same tissue regions in multiple orientations, a single-orientation measurement protocol required correlating susceptibility contrast with fiber orientation from all different regions of the heart. As a result, measurements of anisotropic susceptibility may be affected by the selected specimen orientation. For example, when the heart specimen is oriented with its long axis parallel to the magnetic field, there are likely to be more myocardial fibers that are perpendicular to the magnetic field compared to when the long axis of the heart is perpendicular to the field direction–as it was oriented in this study. Furthermore, bulk magnetic susceptibility shifts due to the presence of spatially varying concentrations of electrolytes, proteins, and other substances with both isotropic and anisotropic susceptibility may yield fluctuations in the single-orientation anisotropy values. Especially large concentrations of diamagnetic electrolytes such as calcium decrease the bulk magnetic susceptibility of that particular tissue region, and have been shown to yield both image phase fluctuations in the brain [12] and decreased signal intensity in fluid-filled phantoms [48]. In a study of the dog heart, concentrations of calcium and other electrolytes in myocardial tissue vary only mildly between the septum and the left ventricle (LV) and right ventricle (RV) walls, as described in Table 3 [49]. Such disparities would result in small bulk isotropic susceptibility differences (<1 ppb) between these two regions and are not sufficient to explain the observed susceptibility variation. Nevertheless, in diseased hearts, *large* decreases in bulk magnetic susceptibility in different tissue regions may be indicative of pathologies associated with abnormal levels of electrolytes, e.g., calcium and iron deposition. Hence, susceptibility and susceptibility anisotropy may potentially be sensitive to diseases correlated with calcium elevation such as cardiac hypertrophy [50].

**Table 3:** Estimated bulk magnetic susceptibility shift differences due to electrolytes in myocardial tissue

Electrolyte	Molar susceptibility^a^ (CGS, cm^3^/mol) [68]	LV concentration^b^ (CGS, mol/cm^3^) [49]	RV concentration^b^ (CGS, mol/cm^3^) [49]	LV–RV Volume susceptibility^c^ difference (SI, unitless)
	χ_*m*_ × 10^−6^	*c* × 10^−6^	*c* × 10^−6^	χ_*v*_, ppb
Ca^2+^	−10.4	0.80 ± 0.10	0.90 ± 0.27	0.01
Cl^−^	−23.4	29.07 ± 2.03	31.53 ± 2.94	0.72
K^+^	−14.9	91.04 ± 4.93	88.78 ± 5.64	−0.42
Mg^2+^	−5.0	10.49 ± 1.49	10.07 ± 1.58	−0.03
Na^+^	−6.8	32.97 ± 1.60	34.94 ± 5.64	0.17

The volume susceptibility differences between the LV and RV walls due to electrolytes are very small compared to the observed susceptibility anisotropy

^a^Magnetic susceptibility values for these electrolytes are typically reported in the literature as molar susceptibilities using CGS units

^b^Calculated from dog myocardium data and the density of fat-free myocardial tissue, 1.054 g/cm^3^ [69]

^c^Conversion from molar to volume susceptibility follows χ_*v*_ = χ_*m*_ *c*. Conversion from CGS to SI units follows χ_*v*_(SI) = 4πχ_*v*_(CGS)

### Other potential sources of anisotropy

The peptide bonds of axially aligned myosin and tropomyosin α-helices, though a significant source of diamagnetic anisotropy, may not act alone in producing orientation-dependent susceptibility contrast in the myocardium. Other potential sources of anisotropy include actin, collagen, hemoglobin, myoglobin, and lipids. Actin represents 25 % of myofilament proteins in normal rodent myocardium (myosin and tropomyosin represent about 50 % and 15 %, respectively) [51]. Both globular and filamentous actin forms contain α-helix structures. However, the α-helices within actin exist in a wide range of orientations relative to the filament axis [34], and birefringence measurements show that actin only weakly aligns to an applied magnetic field [2]. Collagen, another structural protein, may also influence the observed susceptibility anisotropy of the myocardium. Due to the orientation of its peptide bonds, collagen exhibits susceptibility anisotropy that is both directionally opposite and half as strong as that of polypeptide α-helices [18]. However, because collagen constitutes such a small fraction of rodent myocardium, < 2 mg/g [52], relative to myofibrillar proteins, 41.5 ± 1.1 mg/g [53], the susceptibility anisotropy of the thick and thin filament proteins appears to be more dominant.

Hemoglobin and myoglobin may have an effect on myocardial susceptibility anisotropy measurements if the distribution of these proteins in the tissue is non-uniform. For instance, if greater concentrations of paramagnetic deoxyhemoglobin exist in tissue regions with more parallel myofibers, it may increase the measured susceptibility anisotropy. To mitigate any possible effects due to deoxyhemoglobin, the myocardium was extensively perfused with a heparin/saline solution to flush blood out of the tissue during specimen preparation (see *Methods*). Deoxymyoglobin is a paramagnetic globular protein with a large molar susceptibility anisotropy of −9.73 ± 0.38 × 10^−4^ cm^3^/mol [54]. However, myoglobin molecules are not structurally oriented in tissue since they are mobile and diffuse [55]—sliding past one another with little frictional interaction [56]—so collectively they do not produce susceptibility anisotropy, but rather a bulk magnetic susceptibility shift dependent on the mean susceptibility value (1.35 × 10^−2^ cm^3^/mol [54]) and local concentration of myoglobin. In human myocardium, myoglobin content within the LV is quite consistent (including the septum and posterior, lateral, and anterior walls), but greater in the LV (2.4 mg/g wet tissue) than the RV (1.9 mg/g) [57]. Similar to the case of electrolytes (Table 3), the resulting bulk susceptibility difference between the LV and RV walls due to myoglobin is very small, an estimated 0.4 ppb, and is not sufficient to explain the observed susceptibility variation.

Finally, the lipids that form the sarcoplasmic reticulum surrounding individual myofibrils may be a source of susceptibility anisotropy. Lipids are known to be more diamagnetic in the direction parallel to their chain structure, and are believed to be a major source of anisotropy in brain white matter. In rodents, however, the lipid volume fraction is ~0.16 [58] in brain white matter and only ~0.03 in myocardium cells [59]. It is worth noting that the cell volume fraction of myofibrillar mass is ~0.45–0.47 in rodent myocardium [59, 60], further suggesting that that the known anisotropy of myofibrillar peptide bonds is a major contributor, and most likely the dominant contributor, to the orientation-dependent susceptibility contrast visualized with CMR.

## Conclusions

Susceptibility imaging may potentially be used to determine the organization and integrity of myofibers by probing the anisotropic magnetic susceptibility of myocardial tissue. The multi-filament model demonstrates that the arrangement of the diamagnetically anisotropic peptide bonds forming these myofibers contributes substantially to the bulk susceptibility anisotropy of the heart. Greater understanding of the mechanisms generating this anisotropy may lead to improved techniques for studying models of healthy and diseased myocardium.

## Abbreviations

CMR: Cardiovascular magnetic resonance
DTI: Diffusion tensor imaging
GRE: Gradient-recalled echo
LV: Left ventricle
ROI: Region of interest
RV: Right ventricle
SE: Spin echo
SNR: Signal-to-noise ratio
STI: Susceptibility tensor imaging
TE: Echo time
TR: Repetition time
